# Production of Embryonic and Fetal-Like Red Blood Cells from Human Induced Pluripotent Stem Cells

**DOI:** 10.1371/journal.pone.0025761

**Published:** 2011-10-13

**Authors:** Chan-Jung Chang, Koyel Mitra, Mariko Koya, Michelle Velho, Romain Desprat, Jack Lenz, Eric E. Bouhassira

**Affiliations:** 1 Department of Medicine, Hematology, Department of Cell Biology, Albert Einstein College of Medicine, Bronx, New York, United States of America; 2 Department of Genetics, Albert Einstein College of Medicine, Bronx, New York, United States of America; Southern Illinois University School of Medicine, United States of America

## Abstract

We have previously shown that human embryonic stem cells can be differentiated into embryonic and fetal type of red blood cells that sequentially express three types of hemoglobins recapitulating early human erythropoiesis. We report here that we have produced iPS from three somatic cell types: adult skin fibroblasts as well as embryonic and fetal mesenchymal stem cells. We show that regardless of the age of the donor cells, the iPS produced are fully reprogrammed into a pluripotent state that is undistinguishable from that of hESCs by low and high-throughput expression and detailed analysis of globin expression patterns by HPLC. This suggests that reprogramming with the four original Yamanaka pluripotency factors leads to complete erasure of all functionally important epigenetic marks associated with erythroid differentiation regardless of the age or the tissue type of the donor cells, at least as detected in these assays. The ability to produce large number of erythroid cells with embryonic and fetal-like characteristics is likely to have many translational applications.

## Introduction

The development by the Yamanaka group of a method to reprogram somatic cells into induced pluripotent stem cells (iPS) by over expression of pluripotency factors hold considerable promises for the development of stem cell therapies [1]–[5]. In the mouse system, the differentiation potential of iPS has been tested by chimera formation followed by germ line transmission [6]–[8] and more recently by tetraploid complementation [9], [10]. These experiments univocally demonstrate that iPS are very similar to embryonic stem cells since both cell types when placed in the blastocyst environment can differentiate into full term mice. However, several recent reports have shown that the expression profile of iPS is subtly different from that of hES cells[11]–[13] and that iPS might contains genetic mutations induced by the reprogramming process itself. Similarly, the epigenetic profiles of iPS has also been shown to differ from that of ES cells [14]–[20].

Despite these reports, whether ES and iPS are functionally different remains unclear since hESC themselves are quite variable because of their isolation and culture histories and because they carry different genomes. The observation that multiple ES and iPS cell lines can give rise to apparently normal mice suggests that the epigenetic pluripotency program is relatively flexible and that multiple epigenetic states are permissible during early development maybe because reprogramming mistakes or epimutations acquired in culture can be erased during the developmental process. In the case of human iPS, in vivo experiments cannot be performed to determine if a particular iPS clone is appropriately reprogrammed because of obvious ethical reasons. Other means of identifying fully reprogrammed iPS must therefore be developed[21]. One possible approach is to careful examined the *in vitro* differentiation of iPS into well defined cell types and to compare the results with that of hESC.

Human ES cells can easily be differentiated into hematopoietic cells using a variety of methods [22]–[26]. We have previously shown that human ES cells can be differentiated into hematopoietic and red blood cells by co-culture on a feeder layer of immortalized human fetal hepatocytes [23], [27]. Importantly, we found that in this system hESC differentiation closely recapitulates early human erythropoiesis since we observed sequential expression of Hemoglobin Gower1 (ζ_2_ε_2_), Hemoglobin Gower 2 (α_2_ε_2_), and Hemoglobin F (α_2_γ_2_) but that they could produce only very small amounts of Hemoglobin A (α_2_β_2_) [26], [28]–[30]. The proliferation potential of the erythroid progenitors and the morphology of the erythroblast series obtained also mimicked that seen in early development. The subtle switches in globins that we observed in hESC therefore seemed very well suited to assess reprogramming of iPS.

The first goal of the present study was to determine if iPS differentiation into erythroid cells would follow the same patterns as that observed for hESC and resullts in the sequential production of progressively more developmentally mature red cells. The second goal was to determine whether the age of the donors used to produce iPS could influence the type of red cells produced in our system. The third goal of the study was to assess the differentiation potential of iPS into red blood cells because differentiation of iPS into hematopoietic and mature erythroid cells might have major translational applications.

To achieve these goals, we have produced iPS from somatic cells of various ages and induced their differentiation using the approach that we previously published for hESC. We found that it was possible to produce large amounts of erythroid cells from iPS and that the cells produced were very similar in their globin expression patterns to the cells obtained by differentiation of hESC regardless of the age of the donor of the cells. Therefore, reprogramming with the four original Yamanaka pluripotency factors leads to complete erasure of all functionally important epigenetic marks, at least as detectable in our erythroid differentiation system.

## Results

Induced pluripotent stem cells were produced with viruses expressing Oct-4, Sox2, KLF4 and c-Myc generously provided by Dr Yamanaka. Viruses expressing either the human or the mouse version of the four factors were tested. Three somatic cell types were used: embryonic mesenchymal stem cells that were previously derived from hESC [31], [32], fetal liver mesenchymal stem cells and adult skin fibroblasts that were derived in our lab. Exponentially growing cells were infected with retrovirus expressing the four factors and iPS colonies were picked about 2 weeks later based on morphology and expanded.

**Figure 1:**
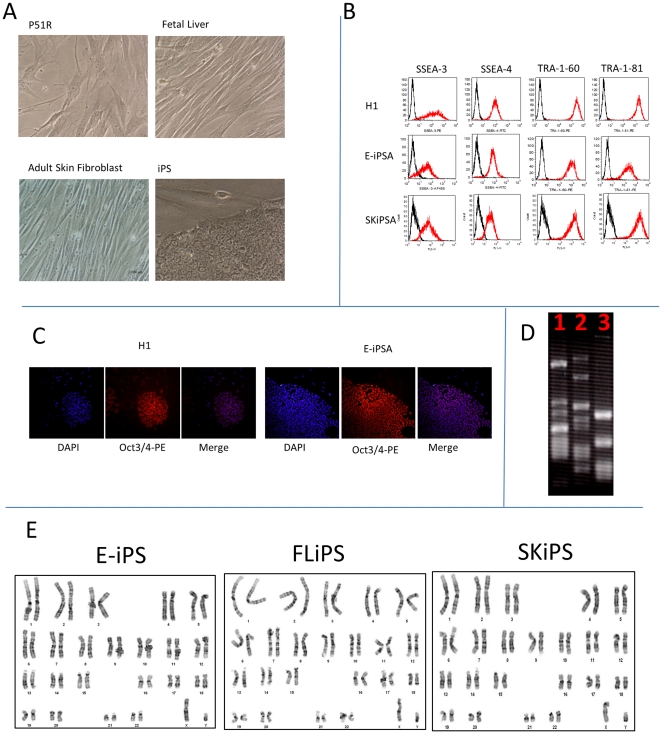
iPS characterization. A: Phase contrast micrograph illustrating the morphology of human iPS cells on Matrigel. B: FACS analysis. Histograms illustrating expression of surface marker, SSEA-3, SSEA-4, Tra-1-60, and Tra-1-8 on ES and iPS cells. The black histograms represent the IgG control. C: immuno-micrographs illustrating expression of transcription factor Oct3/4. Cells were permeabilized and stained with PE-labeled anti-Oct4 antibodies and with DAPI. Most cells in the iPS and the H1 colonies exhibited nuclear Oct3/4 expression D. LM-PCR analysis. Photograph of a 1% agarose gel illustrating the result of an LM-PCR determination of integration site patterns. E: Karyotyping of iPS derived from different cell of origins.

Colonies composed of tightly packed cells with a high nucleo-cytoplasmic ratio and with the typical ES cell morphology could be observed after two to three passages in hESC culture medium (Figure 1A). To ascertain that these cells were indeed reprogrammed to a pluripotent state, we first tested them by flow cytometry using a panel of antibodies that are known to be expressed at high levels in hESC [33]. This analysis revealed that, as expected, most of the colonies obtained expressed high levels of SSEA-3, SSEA4, TRA 1–60 and TRA 1–81(Figure 1B). Further analysis by immuno-cytochemistry using anti Oct-4 antibodies confirmed these studies since most colonies display a homogenous nuclear staining for this ESC-restricted transcription factor (Figure 1C). To characterize these iPS further and to ascertain that individual colonies were indeed clonal, we evaluated the integration patterns of the four reprogramming viruses using LM-PCR from 10 of the iPS that had been derived from embryonic or fetal liver mesenchymal cells. This analysis revealed that many of the colonies that we had picked had different patterns of sites of integration demonstrating that they were derived from independent reprogramming events (Figure 1D and Table S1). These iPSCs clones all had normal karyotyping (Figure 1E).

We then selected independent iPS clones and performed an expression analysis at the transcriptional level. We first analyzed reactivation of the Oct4 and Nanog genes by quantitative RT-PCR and found that the levels observed in the iPS were within 2 to 6 fold the levels found for these genes in H1 and H9 cells when the results were normalized with either GAPDH or β_2_-microglobulin (Figure S1).

**Figure 2:**
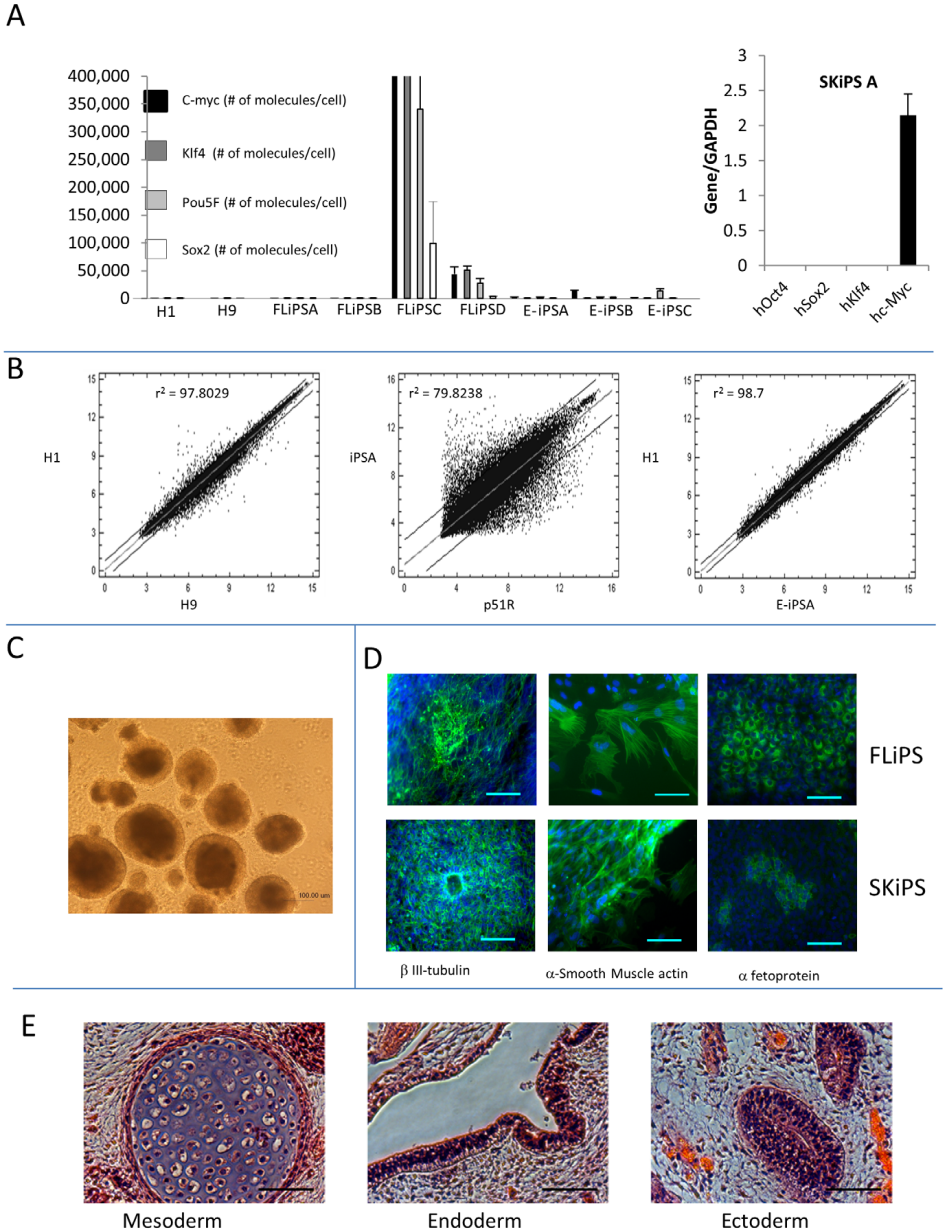
Expression and differentiation analysis. A: Expression of the virally-transduced reprogramming factors cDNA. Y-axis represent absolute number of copies of cDNA per ng of RNA determined using a standard curved based on a purified PCR product. Expression of the transduced cDNA was mostly silent except for FL iPS C (see text). B: Global expression patterns: Scatter-plot illustrating an Affymetrix micro-array analysis of iPS as compared to H1 and to parental somatic cells. Micro-array results were normalized by RMA. Average of two biological replicates is plotted. Left scatter-plot illustrates expression of H1 and H9 cells, middle scatter-plot, expression of iPSA versus p51R parental cells and right scatter plot expression of iPSA versus H1. iPS are undistinguishable from ES cells in this assay. All iPS were at least at passage 20 when the RNA were extracted. C: Embryoid bodies. Phase contrast micrographs illustrating 5 days EB formed from iPS. D: Spontaneous differentiation: Immuno-fluorescence micrographs illustrating iPS differentiation into the three germ layers. Two days EB were transferred to chamber slides containing ES medium without bFGF. After 7 days, the differentiated cells were stained with FITC labeled antibodies against β-III-tubulin (ectoderm), α-smooth muscle actin (mesoderm) and α-feto-protein (endoderm) and counter-stained with DAPI. Results from 2 different iPS are shown. (Bar = 100μm). E: Micrographs illustrating H and E stained slide of teratomas generated from an iPS. iPS can differentiate into teratomas containing the three germ layers (see also Figure S5).

We then assessed the level of expression of the virally transduced cDNA coding for the four pluripotency factors in 7 iPS clones derived either from embryonic (E-iPS) or fetal liver (FL-iPS) mesenchymal cells. The reprogramming factors must be silenced at the end of the reprogramming process for the iPS to be considered fully reprogrammed [1]–[3]. RT-PCR analysis revealed that the iPS clones were not identical (Fig 2A): three clones had undetectable levels of expression of the four factors (E-iPSA, FL-iPSA and FL-iPSB); two clones (E-iPSB and C) respectively exhibited small but detectable levels of the transgenic myc and Oct4); one clone (FL-iPSD) expressed low levels of myc, Klf4 and Oct-4 and interestingly, the virally-transduced four factors had completely escaped silencing in clone FL-IPS C which expressed high levels of all four of the virally-encoded factors.

To assess expression genome-wide, we then performed micro-array experiments in which total RNA from 4 iPS were compared with the H1 and H9 cell lines using Affymetrix Hu_Gene 1.0 arrays. H1 and H9 ES cells were at passage 50 to 80, while the iPS were at passage 5 to 15. Biological replicates were performed for all samples.

As shown in Figure 2B and S2, comparison of the iPS with the two control hESC lines demonstrated that most of the genome of the iPS was completely reprogrammed to an expression pattern typical of pluripotent cells since we found that all the iPS tested were as close to the H1 cell line (coefficient of correlation between 0.990 and 0.995) than H1 is to H9 (r = 0.989). Clustering analysis confirmed these findings since we were unable to get the H1 and H9 cells to cluster away from the iPS in unsupervised analyses.

Interestingly, FL-IPS C, the iPS where the four factors were not silenced, was indistinguishable in this assay from the iPS that had completely silenced their transgenes (Figure S2). We conclude from these experiments that the iPS that we produced with the four factors were undistinguishable from ES cells in their expression patterns, even when silencing of the virally transduced factors is incomplete. Of course we cannot exclude subtle differences between ES cells and iPS that would have been undetected by our micro-arrays.

To further investigate potential differences between the iPS caused by insertional mutagenesis, we examined in details expression of the genes located 200 kb or less from an integration site (Figure S3). Remarkably, we could not detect any effect of the integration of the viruses in any of the iPS tested, even in FL IPS C, the iPS where silencing of the four pluripotency factors did not occur. This is surprising in light of previous studies in which we had shown that randomly inserted transgenes could have dramatic effects on expression of the neighboring genes [34]–[37]. This discrepancy might be linked to the different regulatory elements present in the virus backbone and in the transgenes that were used.

Having shown that we had produced iPS that were undistinguishable from undifferentiated ES cells by several criteria, we examined their differentiation potential via the production of embryoid bodies (EBs) followed by RT-PCR and by immnuno-cytochemistry (Figure 2C and 2D). These studies revealed that most of the iPS tested were able to produce cells from the three germ layers, although some variability was observed between clones. Figure S4 illustrates an RT-PCR analysis of 5 days EB induced to differentiate into mesodermal, ectodermal, and endodermal derivatives using various cytokine cocktails (see methods).

We then tested whether the iPS that we generated could form teratomas containing the three germ layers. As shown in Figures 2E and S5 all clones of iPS tested showed the abilities to form teratoma in NOD-SCID Gamma mice and all teratomas tested were shown to contain cells from the three germ layers after H and E staining.

To determine if iPS derived from embryonic, fetal or adult tissues were able to differentiate into blood derivatives, we co-cultured randomly selected iPS clones for each donor sources with FhB-hTERT fetal hepatocytes since we had previously shown [23], [27], [28] that this procedure efficiently leads to the production of a CD34^+^ cell population which contains a fraction of cells able to differentiate into mature primitive and early definitive erythrocytes. A total 9 iPS lines were tested, three derived from embryonic mesenchymal cells, three from fetal mesenchymal cells and three from adult fibroblasts.

**Figure 3:**
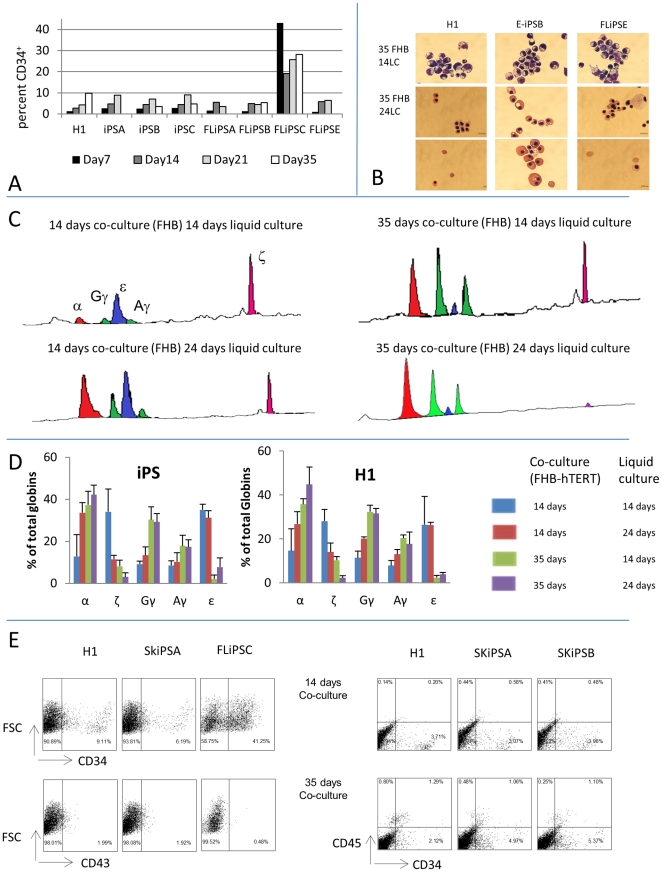
Hematopoietic differentiation. A: Production of CD34 positive cells: hES and iPS cells were co-co-cultured for 7 to 35 days with FHB-hTERT. Percentage of cell expressing CD34 was then assessed by FACS using FITC-labeled anti-CD34 antibodies. Most iPS produced similar level of CD34^+^ cells as H1 except FL iPSC which produced 10 to 15 times more CD34^+^ cells. B: Giemsa staining of erythroid cells obtained by differentiation of H1 or iPS-derived CD34^+^ cells obtained from co-cultures with FHB-hTERT for 35 days. After the co-culture, the cells were placed in liquid culture for 14 days (first row) or 21 days (rows 2 and 3) and cytospins were performed. iPS-derived cells have morphologies very similar to ES-derived cells regardless of the age of the donor. C: Chromatograms illustrating HPLC analyses of globins produced by ES or iPS derived erythrocytes. Top left chromatogram: Erythrocytes produced after 14 days of FHB-hTERT co-culture and 14 days of liquid culture express mostly Hb Gower I (ε_2_ζ_2_), bottom left: after 10 more days of liquid culture (24D), erythroblast down-regulated ζ-globin leading to the production of Hb Gower 2 (ε_2_ζ_2_). Erythroblasts produced after 35 days of co-culture with FH-B-hTERT and 14 days (top right) or 24 days (bottom right) of liquid culture express mostly Hb F (γ_2_α_2_). D: Histograms summarizing HPLC results from 9 iPS clones (left) or from triplicated differentiation experiments of H1 ES cells (right). Erythroid cells generated by co-culture of iPS on FhB-hTERT for 14 days exhibit a major ζ to α globins switching during their maturation from basophilic to orthochromatic erythroblasts in liquid culture. Cell generated after 35 days of co-culture on FhB-hTERT express primarily gamma globins and have therefore switched their expression of the β-like globin genes from ε to γ globin as compared to cells obtained after 14 days of co-culture. Globin expression patterns of iPS-derived cells are very similar to what is observed with hESC. E: Flow cytometry data showing that H1 and SK-iPS after co-culture with FHB-hTERT cells for 14 days cells differentiate into cells that express CD34 and CD43. By contrast, FL-iPS C-derived cells, express CD34 but not CD43. After liquid culture ES and iPS-derived cells differentiate into cells that co-express CD34^+^ and CD45^+^. FL-iPS C do not proliferate.

Figure 3A illustrates a FACS analysis of cells incubated for increasing numbers of days with FHB-hTERT. The yield of CD34^+^ cells in multiple replicated experiments was quite variable since it ranged between 1 and 9% for both the H1 and the iPS lines but no statistically significant differences were observed. It is noteworthy that for both the H1 control and for the various iPS tested, the number of CD34^+^ cells produced increase linearly with time in co-culture. This time dependence of the number of CD34^+^ studies was not detected in our earlier studies [23], [27], [28].

Importantly, FL-IPS C, the iPS which had no silenced the four transgenic factors differed from all other iPS because the percentage of CD 34^+^ cells produced was 10 to 15 times higher than that of the H1 line or of the other iPS (Figure 3A). Therefore, although, the global mRNA expression pattern of this iPS was undistinguishable from that of the other iPS, FL-iPSC has a strikingly different differentiation potential than the other iPS either because of the ubiquitous expression of one or more of the four factors, or because of some other epigenetic abnormality.

To determine if these iPS-derived CD34^+^ cells could differentiate into red blood cells, we cultured them in our 4-step liquid culture system designed to amplify erythroid progenitors and induce their differentiation into mature red blood cells. Again, 9 iPS lines were tested. As shown in Figure 3B and S6, these experiments revealed that CD34^+^ cells derived from iPS co-cultured for 14 or 35 days with FHB-hTERT yielded erythroid cells that did not enucleate and that had a primitive morphology very similar to that of erythroid cells derived from H1 hESC in the same conditions. Quantification of the number of cells confirmed that iPS have an erythroid differentiation potential equivalent to that of H1 cells: Plating of 100,000 hESC or iPS-derived CD34^+^ cells yielded at the end of the liquid culture period varied between 0.5 and 8 million erythroid cells. This relatively large variation could not be reduced despite efforts at standardization of the experimental conditions and likely reflect the complexity of the CD34^+^ cells plated. No statistically significant differences were detected between the iPS and the H1 line. However, because of the experimental variations observed, we cannot exclude that a much larger number of experiments would have revealed subtle differences.

We then performed HPLC analysis on erythroblasts obtained after 14 days and 24 days of liquid culture to determine globin expression patterns since we had previously demonstrated that H1-derived erythroblasts express predominantly Hb Gower 1 after 14 days of liquid culture, and Hb Gower 2, 10 days later. Figures 3C and 3D, which respectively illustrates and summarizes the results for the three embryonic, the three fetal and the three adult iPS tested, demonstrate that iPS-derived erythroblasts undergo the same globin switches during their maturation than H1 cells. We conclude from these experiments that iPS are reprogrammed to an embryonic hematopoietic potential regardless of the age of the donor. To determine if iPS could differentiate into fetal-like red blood cells that can enucleate and express Hb F, we then co-cultured 3 clones of iPS derived from embryonic mesenchymal cells, 3 from fetal liver mesenchymal cells and 2 from adult skin fibroblasts for 35 days with FhB-hTERT, sorted the CD34^+^ cells, differentiated them in liquid culture for 24 days, and performed a morphological and HPLC analysis. This revealed that regardless of the age of the donor, the iPS could differentiate into enucleated red blood cells that contain predominantly Hb F and therefore that they are able to express an erythroid program that is similar to that of hESC (Figure 3C and 3D).

Interestingly, CD 34^+^ cells obtained from 14 or 35 days of co-culture of FL-iPS C with FHB-hTERT were unable to differentiate into erythroid cells. Since the CD34 antigen is expressed on a wide variety of cell types [38]–[40], this result could be explained either by the fact that these cells were not hematopoietic or by the fact that they were hematopoietic but unable to differentiate into the erythroid lineage. Vodyanik et al. [41] have shown that ES cell-derived CD34+ cells that have hematopoietic potential co-express CD43. We therefore assessed CD 43 expression on FL-iPSC-derived CD34+ cells and on 2 iPS clones (derived from adult skin) as well as on H1 derived cells (Figure 3E). These experiments revealed that Fl-iPS C did not express CD 43 suggesting that they are not hematopoietic By contrast, the H1 and the iPS cells tested expressed CD 43 at low level after the co-culture and at higher levels 7 days after the beginning of the liquid culture. The CD45 marker could mostly be detected on cells derived from 35 day co-culture.

This analysis of FL iPS C show that iPS that are undistinguishable from H1 by morphologically, surface antigen, expression profiles and global gene expression pattern can be grossly dysfunctional in their differentiation potential. Whether this is due to over expression of one of the four factors or to another epigenetic anomaly remains to be determined

## Discussion

We have produced human iPS from embryonic, fetal, and adult cells and demonstrated that they were similar to the H1 and to the H9 ES cell lines by morphology, surface antigen expression, global expression pattern and the ability to form the three germ layers in *in vitro* and in vivo differentiation assays. We have also shown that iPS differentiation recapitulates early erythroid differentiation including two different globin switches to the same extent than ES cells. Several groups have shown that iPS can differentiate into erythroid cells[42]–[48]. We characterize here for the first time globin expression patterns by HPLC after differentiation of and show that they are very similar to those observed after hESC differentiation. We therefore conclude that human somatic cells can be fully reprogrammed to a pluripotent state and that they do not retain any functional memory of their previous differentiation and developmental state, at least as could be detected in our assays. These conclusions are based on analysis of embryonic and fetal mesenchymal cells, and of adult fibroblasts. We cannot exclude that iPS produced from other cell types might retain a memory. Others have shown that iPS do retain an epigenetic memory but only at low passages numbers [49], [50].

Analysis of FL-IPS C, The iPS that did not silence the reprogramming viruses revealed that iPS with normal morphology, and expression patterns could be grossly abnormal in their differentiation process. Such iPS might prove a fertile experimental material to better understand the reprogramming process.

Finally, our ability to produce red blood cells with embryonic and fetal phenotypes from iPS might prove useful to study early human erythropoiesis and to test therapies for the hemoglobinopathies. However, our inability to produce adult red blood cells from iPS or ES cells will limit the scope of these experiments to diseases that affect genes expressed in those cells. Development of techniques to produce red blood cells with an adult phenotype from pluripotent embryonic cells is a critical step that remains to be accomplished to unlock the full translational potential of these cells.

## Methods

**Cell culture:** P51R, and fetal Liver mesenchymal cells were grown in DMEM containing 10%FBS, 1 mM L-glutamine (Invitrogen), 1% Penicillin-streptomycin (P/S) and 1% MEM-nonessential amino acids (Invitrogen). Human adult fibroblasts were grown in RPMI-1640 contain 15% FBS, 1 mM L-glutamine (Invitrogen), 1% Penicillin-streptomycin (P/S) amd 1% MEM-nonessential amino acids (Invitrogen).

Human ES cell line H1, H9 and iPS cells were maintained in two different conditions as undifferentiation cells. The first method was by co-culture with p51R mesenchymal cells in Dulbecco modified Eagle medium (DMEM)/F12 media (Invitrogen) supplemented with 20% Knockout Serum Replacer (Invitrogen), 4 ng/mL Basic Fibroblast Growth Factor (bFGF, ProsSpecTany), 1 mM L-glutamine, 1% Penicillin –streptomycin (P/S) [31]. The second method was on matrigel (BD) in DMEM)/F12 media (Invitrogen) supplement with N2B27 medium, 0.5% Bovine Serum Albumin (Sigma), 1 mM L-glutamine, 1% Penicillin –streptomycin (P/S), 100 ng bFGF [51].

**Retrovirus Production:** All virus used in this study are retrovirus. Retroviral expression plasmids containing the mouse [1] or humans [2] 4 pluripotent genes, Oct4, Sox2, Klf4 and c-Myc were obtained from Addgene. One day before transfection, PhoenixAmpho packaging cells (Orbigen) were plated at a density of 2×10^6^/ml in 10 cm cell culture dish with DMEM contain 10% FBS. One day later, 10 ug of plasmid was transfected using lipofectamine 2000 (Invitrogen) for 5 hours at 37°C. After 5 hours, the supernatant was replaced with fresh medium contain 10% FBS for another 12∼24 hours. Supernatant was collected, filtered with 0.45 μm filter and store in −80°C.

**Virus infection.** Exponentially growing cells were infected with the four factors in the presence of 6 ug/ml Polybrene(sigma-Aldrich). After 24 hours, supernatant was discarded and replaced with fresh DMEM medium with 10% FBS. Infected cells were incubated for 5 days at 37°C in 5% CO2, dissociated with trypsin-EDTA (Invitrogen) and transferred to 6 well plates pre-coated with Matrigel containing DMEM and 10% serum. A day after the medium was replaced with DMEM)/F12 media (Invitrogen) supplemented with N2B27 medium, 0.5% human serum albumin (Sigma), 1 mM L-glutamine, 1% Penicillin –streptomycin (P/S), 100 ng bFGF. Medium was then changed every other day until iPS colonies were picked.

**Differentiation of hESC/iPS to CD34+ cells:** Undifferentiated hESC/iPS cells on feeder plates were dissociated with collagenase type IV (1 mg/mL; invitrogen), transferred onto irradiated FH-B-hTERT feeder layers with DMEM supplemented with 20% FBS (Invitrogen), 2 mM L-glutamine, 1% MEM-nonessential amino acids, 100 u/mL penicillin and 100 µg/mL streptomycin. The medium was changed every 2 to 3 days. For 35 days co-culture, cells were plated onto new feeder layers as above. On day 14, 21, or 35 of co-culture, differentiated H1/iPS cells on FH-B-hTERT were dissociated into single cell suspensions by using collagenase IV followed by trypsin/EDTA or Tryple Express (Invitrogen) supplemented with 5% chick serum. CD34 cell separations were performed using EasySep CD34 magnetic beads according to the manufacturer's instructions (StemCell Technologies, Vancouver, BC).

**Amplification and differentiation of CD34^+^ cells.** CD34^+^ cells obtained either from hESCS or from iPS then seeded in the following 4-step system: In the first step (amplification of progenitors), sorted CD34^+^ cells were placed on a 12-well plate at a density of 50,000 cells/mL with serum-free basal medium StemSpan (StemCell Technologies) supplemented with Hydrocortisone (10^–6^ M), IL3 (13 ng/mL), BMP4 (13 ng/mL), Flt3L (33 ng/mL), SCF (100 ng/mL), and EPO (2.7 U/mL) for 7 days. In the second step (differentiation of progenitors to erythroid lineage), the cells were transferred to StemSpan medium supplemented with hydrocortisone (10^–6^ M), IL3 (13 ng/mL), BMP4 (13 ng/mL), SCF (40 ng/mL), EPO (3.3 U/mL) and IGF-1 (40 ng/mL) for 7 days. Cell density was kept below one million cells per mL at all time by adding fresh medium every 2 or 3 days as needed.

**HPLC:** Erythroid cells were collected at different time points from the liquid cultures, washed twice with PBS, and lysed in water by 3 rapid freeze-thaw cycles. Debris were eliminated by centrifugation at 16 000 *g* and the lysates stored in liquid nitrogen before HPLC analysis. HPLC were performed as described [28].

**Flow Cytometry:** Cells were dissociated with trypsin/EDTA, washed, and resuspended in PBS with 1% FBS. The cells (1×10^5^/ml) were then stained for 30 minutes on ice with saturating amounts of fluorescein isothiocyanate (FITC)-conjugated or phycoerythrin (PE)-conjugated monoclonal antibodies. Antibodies recognizing the following human antigens were used: CD34,CD43,CD235a, SSEA-4, (BD Pharmingen) and TRA-1-60, TRA-1-81, SSEA-1, SSEA-4 (eBioscience).

All analyses were done on a Becton Dickinson FACSCalibur laser flow cytometric system (BD Biosciences, San Jose, CA, http://www.bdbiosciences.com) equipped with a Macintosh PowerMac G5 personal computer (Apple Computer, Inc., Cupertino, CA, http://www.apple.com) using CellQuest Pro software (BD Biosciences). Antibodies are described in details in Table S2.

**Immunofluorescence.** Cells were grown on chamber slide coated with matrigel (BD), fixed with 4% paraformadehyde for 10 minutes at room temperature. Cells were permeabilized with 0.1% Saponin(Sigma) in PBS for 10 minutes, stained with PE-conjugated antibody against human Oct3/4 (BD Pharmingen), or with unlabelled α-fetoprotein (R&D system), β-III-Tubulin or α-smooth muscle actin. FITC-conjugated secondary antibodies were used. Labelled slides were mounted in Prolong Gold antifade prior to visualization under a fluorescence microscope (ZEISS, AxioVert 200M). All antibodies are described in Table S3.

**Cytospin and Giemsa staining:** Cells were spun onto poly-lysine–coated slides using a Cytospin 2 apparatus (Thermo Shandon, Pittsburgh, PA). After drying for a minute, slides were stained with Wright-Giemsa reagents (Hema 3 stain; Fisher Scientific, Pittsburgh, PA) following the manufacturer's instructions. Images were processed using ACT-2U version 1.6 software (Nikon, Tokyo, Japan).

**Teratoma Formation.** iPS cells were obtained from feeder cell plates by collagenase IV treatment (1 mg/mL). The iPS colonies were cut with a glass pipette into small clumps. The clumps were collected after they had settled to the bottom of the tube for 5–10 minutes and resuspended with ES medium (100 uL/wells of six well plate). The cell suspension was mixed with one-third the volume of matrigel (BD Bioscience) and 10^6^ cells were injected intramuscularly into the hind leg of a 6–8 week old NSG (NOD.Cg-Prkdcscid Il2rgtm1Wjl/SzJ) mice. After 6–12 weeks, the visible tumors were dissected out and fixed in 10% formalin solution overnight. The tissues were paraffin embedded, sectioned and stained with hematoxylin/eosin using standard procedure.

**RNA extraction, cDNA preparation, and RT-PCR.** Total RNA was isolated (RNeasy Mini Kit, QIAGEN) with DNA elimination (RNase-Free DNase Set, QIAGEN). The RNA was then ran on gel electrophoresis to assess for purity and was then reverse transcribed to cDNA (SuperScript III First-Strand Synthesis System for RT-PCR, Invitrogen). Gene expression was assessed using quantitative real-time PCR (Applied Biosystems, 7900HT). PCR reactions were run in duplicates using a Qiagen SYBR Green Kit. Controls with no primers and no template cDNA were run for each PCR reaction. A control with no reverse transcription was also run to ensure there was no genomic DNA contamination. Gene expression was determined by relative quantification with values corrected for GAPDH or B2M and normalized relative to control cell. Primer sequences are described in supplementary methods(Table S4 and S5).

**Micro array analysis.** Total RNA were hybridized to Affymetrix HUGene ST 1.0 arrays. Data were normalized by RMA using Affymetrix software and analyzed using Statgraphics Centurion 15.2 (Statpoint Inc, Warrenton, VA) and Cluster 3.0 [52]. All data are MIAME compliant and can be downloaded from NCBI GEO archives (GSE26946).

## Supporting Information

Figure S1 **Endogenous pluripotency factors expressed in ES or iPS cells.** Histograms illustrating a Q-RT-PCR analysis of expression of the Pou5F (Oct4) and Nanog in control H1 and H9 cells in Fetal liver mesenchymal cells (FL) in embryonic mesenchymal cells (p51R) and in 7 iPS derived either from fetal liver (FL-iPS A to D), from p51R (iPS A through C) or from skin fibroblast (sk-iPS A). All iPS expressed detectable levels of the 2 endogenous transcription factors. Y axis represents the ratio of expression of the factors compared with H1 as determined by the Delta Ct method.
(TIF)

Figure S2 **Comparison of the transcriptome of the H1 and H9 cell lines with that of four different iPS.** Comparison of the transcriptome of the H1 and H9 cell lines with that of four different iPS. Biological replicates of each sample were hybridized on Affymetrix Hu_Gene 1.0 arrays and the results were normalized by RMA. Average of the biological replicates are plotted.
(TIF)

Figure S3 **Logarithm of expression of genes close to integration sites of transduced reprogramming factors.** Histograms representing expression (determined by Affymetrix array) of all the genes located within 300 kb of 4 of the 8 integration sites that we characterize in iPSA (white bars) as compared to H1 (black bars). No significant differences could be detected in more than 50 integration site analyzed in 4 different iPS suggesting that the integrated viruses had minimal effects.
(TIF)

Figure S4 **Forced differentiation.** iPS were differentiated by EB production followed by incubation for 5 days in either 100 ng/BMP4; wnt3A, ActivinA and 0.5% FBS or 1 uM retinoic acid, 100 ng Noggin; RNA was then extracted and Q-RT PCR were performed. Increase in expression of marker for the three germ layers was detectable for almost all iPS. The level of induction varied from experiment to experiments.
(TIF)

Figure S5 **Teratoma formation of adult Skin iPS cells.** SkiPS cells formed teratoma in the NOD/SCID mice, showed the pluripotency of iPS cells from adult skin fibroblasts. A: the size of the teratoma was around 1 cm. B: the H&E staining showed that the teratomas contain cells derived from the three germ layers.
(TIF)

Figure S6 **Erythroid cells differentiation of HSC derived from SKiPS cells.** Hematopoietic cells derived from iPS after 14 or 35 days of co-culture with FhB-hTERT and 14 and 24 day of liquid culture showed different morpholgies after Giemsa staining.
(TIF)

Table S1 **Integration sites of four factors in iPS.** Integration sites were identified by inverse-PCR.
(DOCX)

Table S2 **Antibodies used for FACS analysis.**
(DOCX)

Table S3 **Antibodies used for Immunocytochemistry.**
(DOCX)

Table S4 **RT-PCR primers for virus genes.** For each gene, top row is forward primer, bottom row is reverse primers. Sizes of the amplicons are in bp.
(DOCX)

Table S5 **RT-PCR primers for endogenous genes.** For each gene, top row is forward primer, bottom row is reverse primers. Sizes of the amplicons are in bp.
(DOCX)
