# Supplementary figures and images for: Production of Embryonic and Fetal-Like Red Blood Cells from Human Induced Pluripotent Stem Cells
Source: PLoS One. 2011 Oct 13;6(10):e25761. doi: 10.1371/journal.pone.0025761 (PMC3192723; doi:10.1371/journal.pone.0025761)

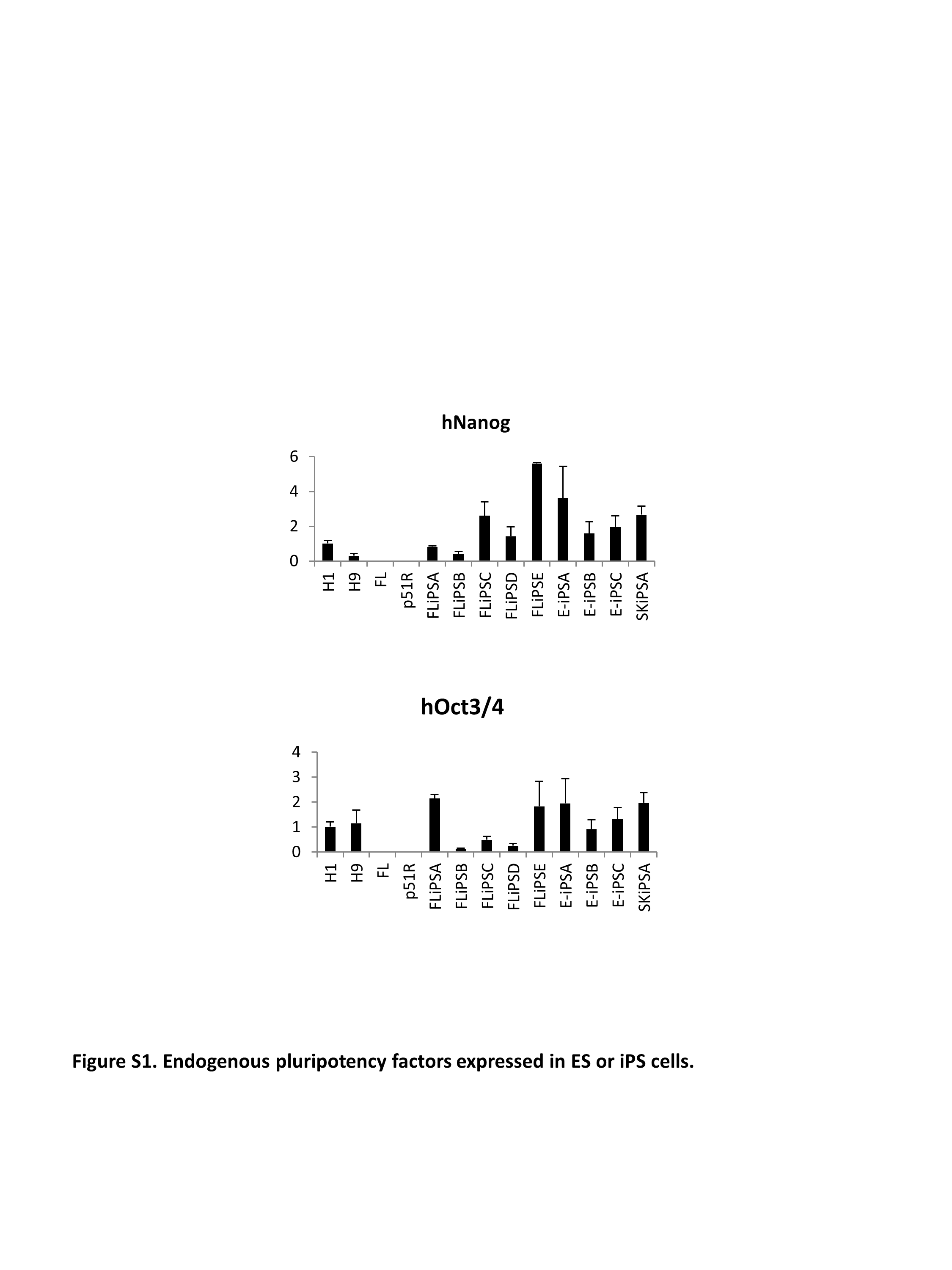

Supplement: Figure S1 — Endogenous pluripotency factors expressed in ES or iPS cells. Histograms illustrating a Q-RT-PCR analysis of expression of the Pou5F (Oct4) and Nanog in control H1 and H9 cells in Fetal liver mesenchymal cells (FL) in embryonic mesenchymal cells (p51R) and in 7 iPS derived either from fetal liver (FL-iPS A to D), from p51R (iPS A through C) or from skin fibroblast (sk-iPS A). All iPS expressed detectable levels of the 2 endogenous transcription factors. Y axis represents the ratio of expression of the factors compared with H1 as determined by the Delta Ct method. (TIF) [file pone.0025761.s001.tif]

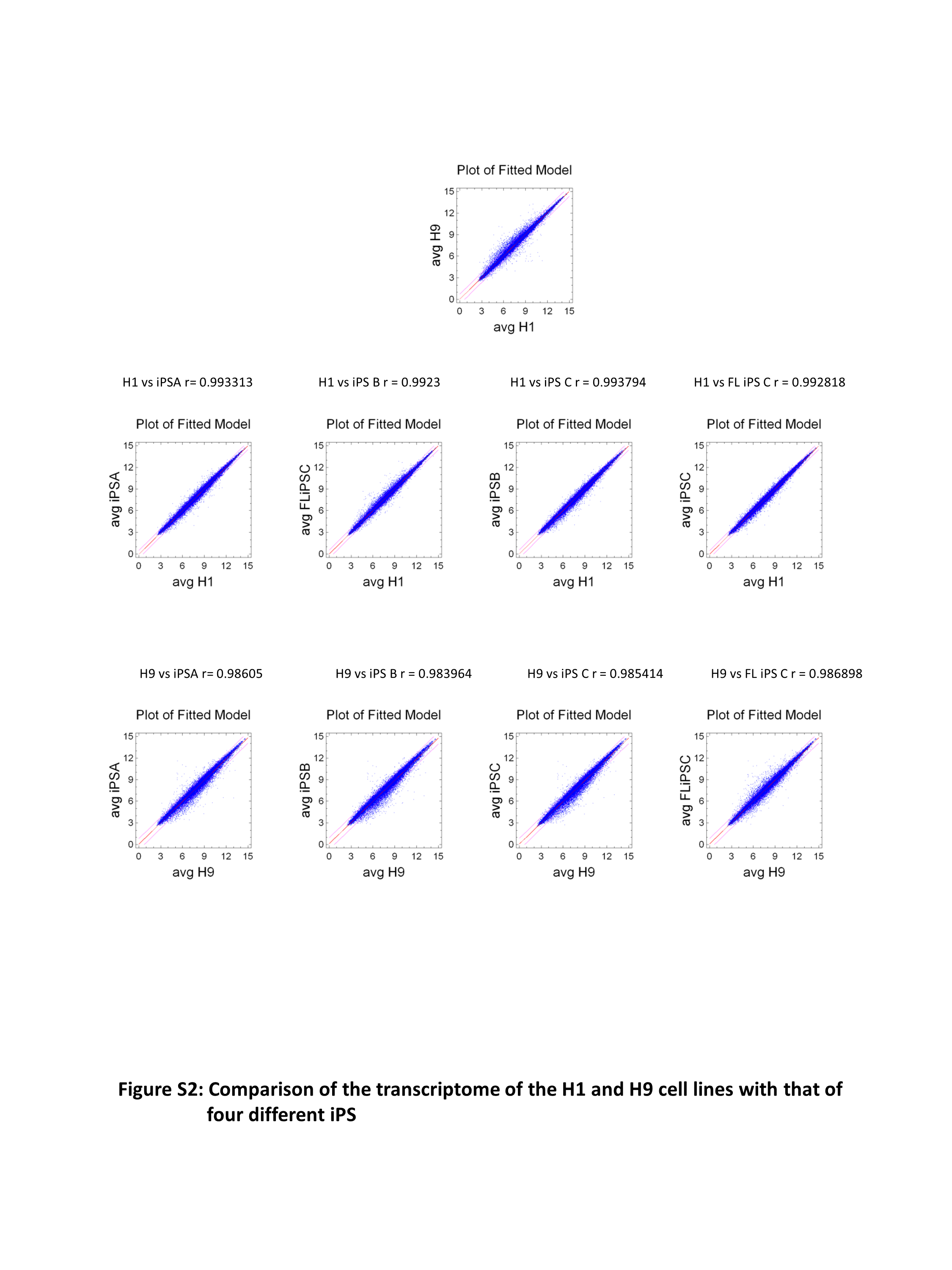

Supplement: Figure S2 — Comparison of the transcriptome of the H1 and H9 cell lines with that of four different iPS. Comparison of the transcriptome of the H1 and H9 cell lines with that of four different iPS. Biological replicates of each sample were hybridized on Affymetrix Hu_Gene 1.0 arrays and the results were normalized by RMA. Average of the biological replicates are plotted. (TIF) [file pone.0025761.s002.tif]

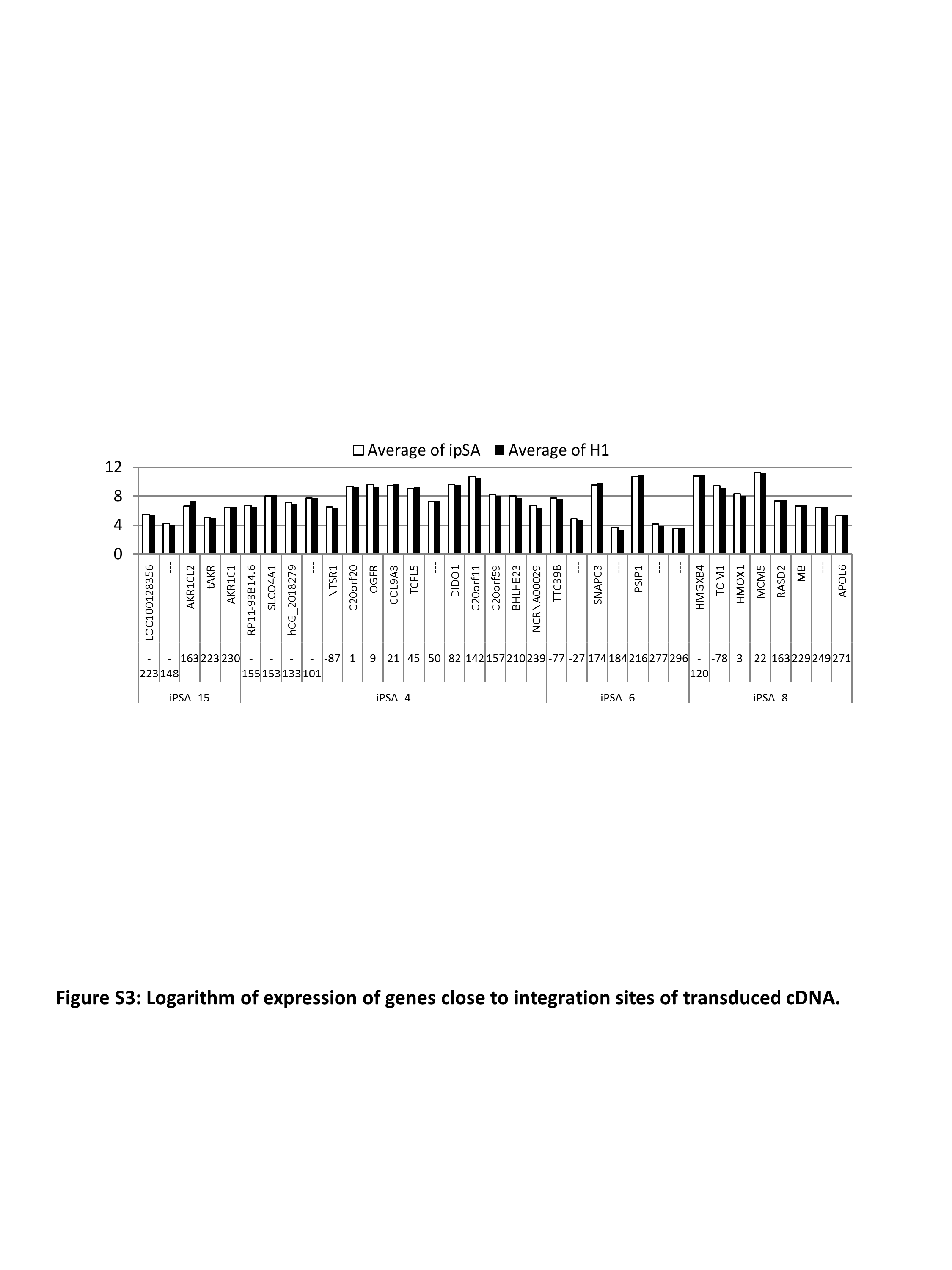

Supplement: Figure S3 — Logarithm of expression of genes close to integration sites of transduced reprogramming factors. Histograms representing expression (determined by Affymetrix array) of all the genes located within 300 kb of 4 of the 8 integration sites that we characterize in iPSA (white bars) as compared to H1 (black bars). No significant differences could be detected in more than 50 integration site analyzed in 4 different iPS suggesting that the integrated viruses had minimal effects. (TIF) [file pone.0025761.s003.tif]

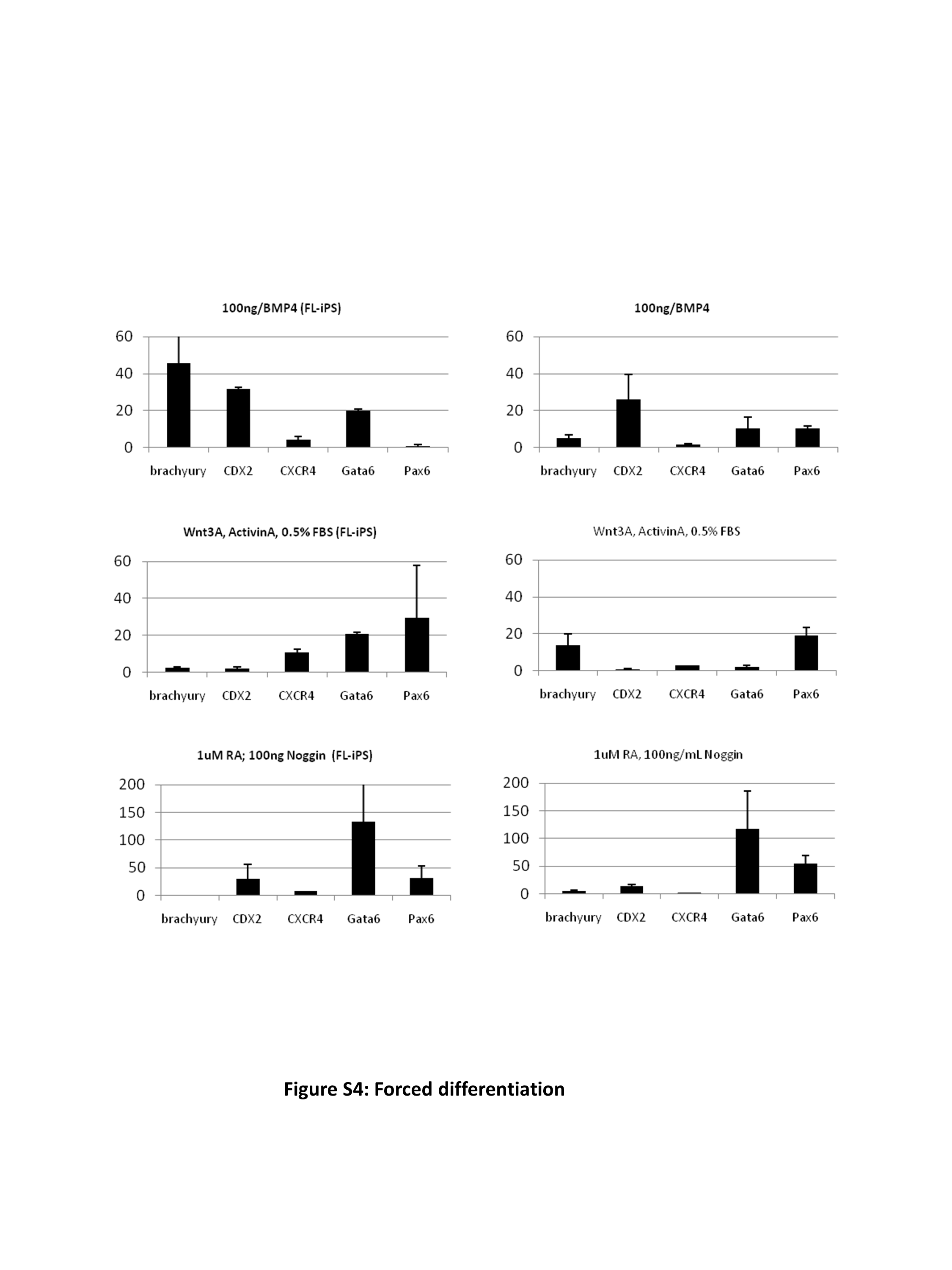

Supplement: Figure S4 — Forced differentiation. iPS were differentiated by EB production followed by incubation for 5 days in either 100 ng/BMP4; wnt3A, ActivinA and 0.5% FBS or 1 uM retinoic acid, 100 ng Noggin; RNA was then extracted and Q-RT PCR were performed. Increase in expression of marker for the three germ layers was detectable for almost all iPS. The level of induction varied from experiment to experiments. (TIF) [file pone.0025761.s004.tif]

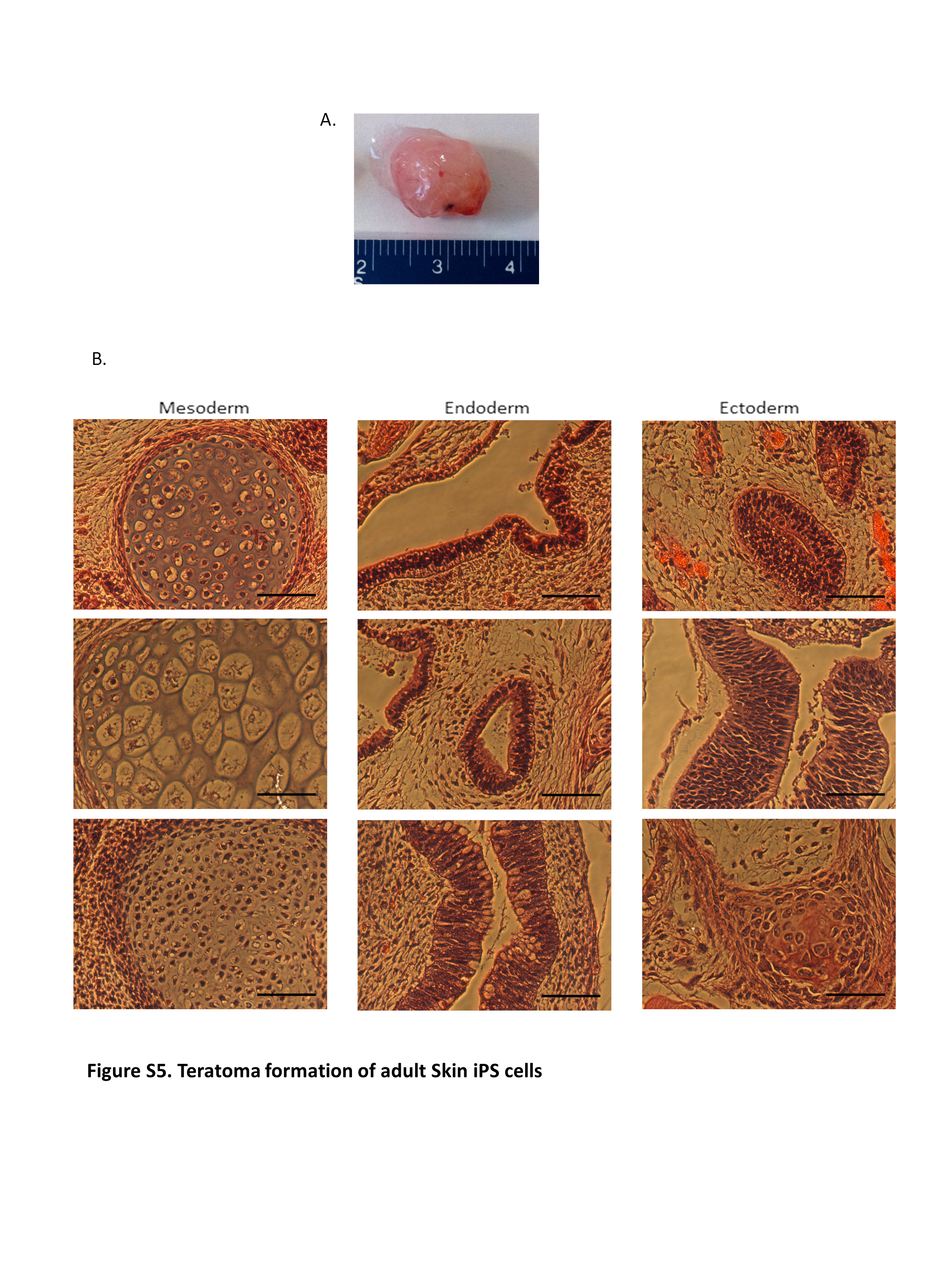

Supplement: Figure S5 — Teratoma formation of adult Skin iPS cells. SkiPS cells formed teratoma in the NOD/SCID mice, showed the pluripotency of iPS cells from adult skin fibroblasts. A: the size of the teratoma was around 1 cm. B: the H&E staining showed that the teratomas contain cells derived from the three germ layers. (TIF) [file pone.0025761.s005.tif]

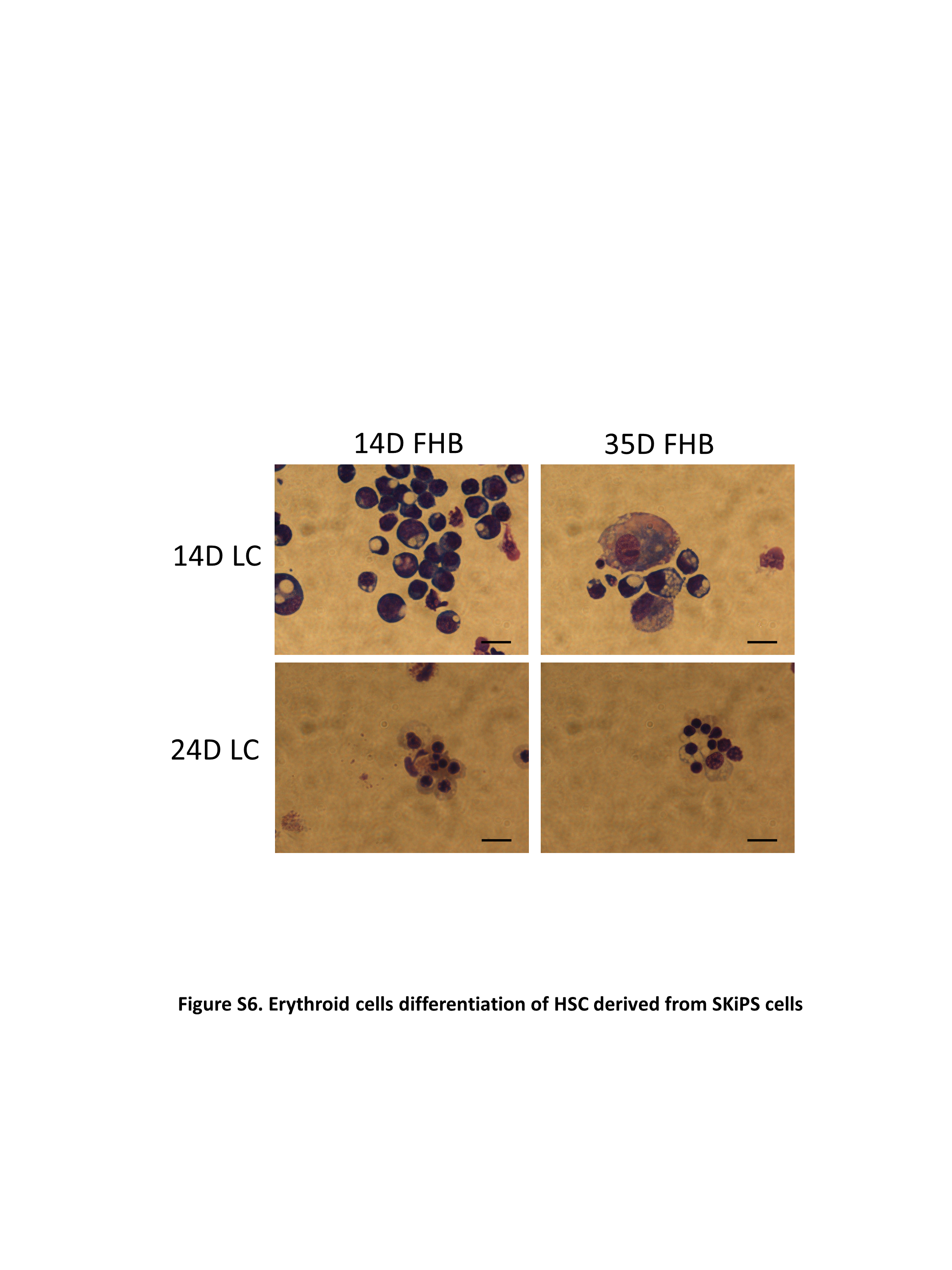

Supplement: Figure S6 — Erythroid cells differentiation of HSC derived from SKiPS cells. Hematopoietic cells derived from iPS after 14 or 35 days of co-culture with FhB-hTERT and 14 and 24 day of liquid culture showed different morpholgies after Giemsa staining. (TIF) [file pone.0025761.s006.tif]
