# Supplementary material for: Production of Embryonic and Fetal-Like Red Blood Cells from Human Induced Pluripotent Stem Cells
Source: PLoS One. 2011 Oct 13;6(10):e25761. doi: 10.1371/journal.pone.0025761 (PMC3192723; doi:10.1371/journal.pone.0025761)
Supplement: Table S1 — Integration sites of four factors in iPS. Integration sites were identified by inverse-PCR. (DOCX) [file pone.0025761.s007.docx]

Table S1: integration sites of four factors in iPS

| **iPS clone** | **Retroviral insertion site** | **Chr** | **nearest gene** | **relative distance; transcriptional orientation** | **nearest genes flanking opposite viral end** | **relative distance; transcriptional orientation** |
| --- | --- | --- | --- | --- | --- | --- |
| E-iPSA | 30598905 | 22 | LIF | 43.9kb, 3' Inverse | HORMAD2 | 122.5 kb, 3' Same |
|  | 61426527 | 20 | C20orf20 | 1.3kb, 5' Inverse | NTSR1 | 86.3 kb, 3' Inverse |
|  | 15247790 | 9 | TTC39B | 59.5kb, Intron, Same | - | - |
|  | 35773364 | 22 | HMOX1 | 3.7kb, 5' Same | TOM1 | 78.1 kb, 3' Same |
|  | 74698650 | 2 | MRPL53 | 1.3kb, 3' Same | MOGS | 6.1 kb, 5' Same |
|  | 13141304 | 9 | MPDZ | 109.1kb, Intron, Same | - | - |
|  | 4704601 | 10 | LOC338588 | 6.3 kb, Exon, Inverse | - | - |
|  |  |  |  |  |  |  |
| E-iPSB | 38699669 | 17 | *CCR7 | 22.1 kb, 3' Same | TNS4 | 41.8kb, 5' Same |
|  | 171052576 | 1 | FMO3 | 7.4 kb, 5' Inverse | C1orf129 | 148 kb, 3' Inverse |
|  | 45456480 | 20 | EYA2 | 66.8 kb, 5' Same | SLCA10 | 118.2kb, 3' Same |
|  | 37319802 | 5 | NUP155 | 51.1 kb, Intron, Same | - | - |
|  | 116924394 | 7 | WNT2 | 38.9 kb, Intron, Same | - | - |
|  | 38462639 | 1 | FHL3 | 8.6 kb, 3‘ UTR, Inverse | - | - |
|  | 105110337 | 10 | PCGF6 | 0.6 kb, Intron, Same | - | - |
|  |  |  |  |  |  |  |
| E-iPSC | 44915047 | 21 | C21orf84 | 16.9 kb, 5' Same | HSF2BP | 164.3 kb, 3’ Same |
|  | 56642147 | 16 | MT2A | 0.3 kb, 5' Inverse | MT3 | 18.9kb, 3' Inverse |
|  | 38334349 | 2 | C2orf58 | 23.9 kb, 5' Same | CYP1B1 | 31 kb, 5' Inverse |
|  | 100143952 | 7 | AGFG2 | 7.1 kb, Intron, Same | - | - |
|  |  |  |  |  |  |  |
| FL-iPSA | 96328152 | 4 | UNC5C | 142 kb, Intron, Inverse | - | - |
|  | 223888319 | 1 | CAPN2 | 1 kb, 5' Same | CAPN8 | 34.9 kb, 5' Inverse |
|  | 97795592 | 1 | DPYD | 591 kb, Intron, Same | - | - |
|  | 109058509 | 6 | ARMC2 | 111.1 kb, 5' Same | FOXO3A | 177.5 kb, 3' Same |
|  | 124683931 | 8 | KLHL38 | 18.7 kb, 5' Same | ANXA13 | 65.7 kb, 3' Same |
|  | 65265625 | 11 | *SCYL1 | 26.9 kb, 5' Inverse | FRMD8 | 111.6 kb, 3' Inverse |
|  | 28503822 | 16 | CLN3 | 0.4 kb, 5' Same | APOB48R | 2.2 kb, 5' Inverse |
|  |  |  |  |  |  |  |
| Fl-iPSC | 68345268 | 16 | PRMT7 | 0.3 kb, Intron, Same | - | - |
|  | 148550862 | 5 | ABLIM3 | 29.8 kb, Intron, Inverse | - | - |
|  | 51787527 | 12 | GALNT6 | 2.3 kb, 5’ Inverse | SLC4A8 | 31.1 kb, 5’ Same |
|  | 42349219 | 8 | SLC20A2 | 47.8 kb, Intron, Same | - | - |
|  | 14109590 | 5 | TRIO | 34.2 kb, 5’ Same | DNAHC5 | 165 kb, 5’ Inverse |
|  | 26522350 | 6 | HCG11 | 0.4 kb, Exon, Same | - | - |
|  |  |  |  |  |  |  |
| FL-iPSD | 43686962 | 19 | PSG5 | 3.7 kb, Intron, Inverse | - | - |
|  | 3506225 | 6 | GPSM3 | 4.8 kb, 3’ Inverse | PBX2 | 122.4 kb, 5’ Inverse |
|  | 103765793 | 8 | KLF10 | 97.8 kb, 5’ Same | AZIN1 | 110.6 kb, 3' Same |
|  | 25056121 | 22 | POM121L10P | 1 kb, 5' Inverse | PIWIL3 | 114.6 kb, 3’ Inverse |
|  | 68318753 | 8 | ARFGEF1 | 62.8 kb, 5' Inverse | CPA6 | 339.9 kb, 3’ Inverse |
|  | 67475332 | 8 | MYBL1 | 50.1 kb, 3' UTR, Inverse | - | - |
|  | 11357216 | 19 | DOCK6 | 15.9 kb, Intron, Inverse | - | - |
|  |  |  |  |  |  |  |
| FL-IPSE | 80178778 | 12 | †PPP1R12A | 150.2 kb, Intron, Inverse | - | - |
|  | 80584309 | 12 | PTPRQ | 253.8 kb, 5’ Inverse | †PPP1R12A | 255.3 kb, 5’ Same |
|  |  |  |  |  |  |  |
| c1 | 81161227 | 4 | FGF5 | 26.5 kb, 5' Inverse | PRDMT8 | 54.8 kb, 3' Inverse |
|  | 52452073 | 15 | GNB5 | 20.1 kb, Intron, Inverse | - |  |
|  | 147515777 | 6 | STXBP5 | 9.7 kb, 5’ Inverse | C6ORF103 | 595.6 kb, 3' Inverse |
|  | 17540165 | 20 | BFSP1 | 0.6 kb, 5’ Same | DSTN | 10.4 kb, 5' Inverse |
|  | 5702420 | 9 | KIAA1432 | 73.1 kb, Intron, Same | - |  |
|  | 22596114 | 16 | LOC653786 | 39.1 kb, 3' Same | HS3ST2 | 229.7 kb, 5’ Same |
|  | 5594341 | 10 | CALML3 | 27.4 kb, 3' Same | ASB13 | 114.2 kb, 3' Inverse |
|  | 87692021 | 1 | LMO4 | 102.1 kb, 5’ Inverse | LOC339524 | 96.6 kb, 3’ Inverse |
|  | 114089615 | 1 | MAGI3 | 156.1 kb, Intron, Inverse | - | - |
|  | 67426144 | 15 | *SMAD3 | 68 kb, Intron, Inverse | - | - |
|  | 21779975 | 16 | LOC730092 | 50.5 kb, 3’ Inverse | OTOA | 90.1 kb, 3’ Same |
|  | 67066432 | 15 | SMAD6 | 71.8 kb, Intron, Same | - | - |
|  |  |  |  |  |  |  |
| c2-2 | 98376415 | 7 | TMEM130 | 91.3 kb, 3’ Inverse | NPTX2 | 129.8 kb, 3’ Same |
|  | 30729544 | 2 | LCLAT1 | 59.4 kb, Intron, Inverse | - | - |
|  |  |  |  |  |  |  |
| c4-1 | 99187746 | 10 | PGAM1 | 1.7 kb, Intron, Inverse | - | - |
|  |  |  |  |  |  |  |
| c4-2 | 94670847 | 11 | CWC15 | 35.9 kb, 3’ Same | AMOTL1 | 169.3 kb, 3’ Inverse |
|  | 96309212 | 2 | TRIM43 | 51.4 kb, 3’ Same | LOC729234 | 367.1 kb, 5’ Same |
|  | 16953860 | 22 | CCT8L2 | 119.8 kb, 3’ Inverse | OR11H1 | 504.1 kb, 5’ Inverse |
|  |  |  |  |  |  |  |
| c8 | 33760820 | 7 | BMPER | 184.3 kb, 5' Inverse | BBS9 | 591.7 kb, 3' Inverse |
|  | 48898880 | X | TFE3 | 2.1 kb, Intron, Inverse | - | - |
|  | 177937360 | 4 | VEGFC | 223.5 kb, 5' Same | NEIL3 | 293.6 kb, 5’ Inverse |
|  |  |  |  |  |  |  |
| c11 | 19743435 | 7 | TWISTNB | 5.2 kb, Intron, Inverse | - | - |
|  | 4528889 | 20 | PRNP | 137.9 kb, 5' Same | ADRAID | 299.2 kb, 5‘ Inverse |
|  | 21299283 | 1 | EIF4G3 | 204.1 kb, Intron, Inverse | - | - |
|  | 36954815 | 17 | PIP4K2B | 1.3 kb, Intron, Inverse | - | - |
|  | 41937221 | 4 | TMEM33 | 0.1 kb, 5’ UTR, Same | - | - |
|  | 2980800 | 18 | LPIN2 | 31.2 kb, Intron, Inverse | - | - |
|  |  |  |  |  |  |  |
| c14 | 73639915 | X | SLC16A2 | 1.2 kb, 5’ Inverse | ZCCHC13 | 115.9 kb, 3’ Inverse |
|  |  |  |  |  |  |  |
| c16 | 61597083 | 11 | FADS2 | 1.37, Intron, Same | - | - |
|  | 107705746 | 3 | LOC28520 | 103.7 kb, 3’ Inverse | CD47 | 104.2 kb, 3’ Same |
