# Supplementary material for: Production of Embryonic and Fetal-Like Red Blood Cells from Human Induced Pluripotent Stem Cells
Source: PLoS One. 2011 Oct 13;6(10):e25761. doi: 10.1371/journal.pone.0025761 (PMC3192723; doi:10.1371/journal.pone.0025761)
Supplement: Table S2 — Antibodies used for FACS analysis. (DOCX) [file pone.0025761.s008.docx]

**Table S2: antibodies used for FACS analysis**

| TRA-1-81 | mIgM | PE | eBioscience | 12-8883-82 |
| --- | --- | --- | --- | --- |
| TRA-1-60 | mIgM | PE | eBioscience | 12-8863-82 |
| SSEA-4 | mIgG3 | FITC | BD Pharmingen | 560126 |
| SSEA-1 | mIgM | PE | eBioscience | 12-8813-73 |
| SSEA-3 | rIgM | AF488 | eBioscience | 53-8833-73 |
| CD34 | mIgG1 | APC | BD Pharmingen | 555824 |
