# Supplementary material for: Production of Embryonic and Fetal-Like Red Blood Cells from Human Induced Pluripotent Stem Cells
Source: PLoS One. 2011 Oct 13;6(10):e25761. doi: 10.1371/journal.pone.0025761 (PMC3192723; doi:10.1371/journal.pone.0025761)
Supplement: Table S3 — Antibodies used for Immunocytochemistry. (DOCX) [file pone.0025761.s009.docx]

**Table S3: antibodies used for Immunocytochemistry**

| bIII Tubulin | mIgG2A | R&D | MAB1195 |
| --- | --- | --- | --- |
| ha-fetoprotein | mIgG1 | R&D | MAB1369 |
| ha-smooth msucle actin | mIgG2A | R&D | MAB1420 |
| Anti-Oct3/4-PE | mIgG1 | BD Pharmingen | 560186 |
| Goat anti-mIgG-AF488 |  | Invitrogen | A11001 |
