# Supplementary material for: Production of Embryonic and Fetal-Like Red Blood Cells from Human Induced Pluripotent Stem Cells
Source: PLoS One. 2011 Oct 13;6(10):e25761. doi: 10.1371/journal.pone.0025761 (PMC3192723; doi:10.1371/journal.pone.0025761)
Supplement: Table S4 — RT-PCR primers for virus genes. For each gene, top row is forward primer, bottom row is reverse primers. Sizes of the amplicons are in bp. (DOCX) [file pone.0025761.s010.docx]

**Table S4: RT-PCR primers for virus genes**

| pMXs | Amplicon | CCAGTGTGGTGGTACG |
| --- | --- | --- |
| vmKlf4 | 173 | CCAACGGTTAGTCGGG |
| vmSox2 | 166 | GGGCTGTTCTTCTGGTTG |
| vmOct4 | 183 | CTGGAGGCCCTTGGAA |
| vmc-Myc | 144 | CGCAGATGAAATAGGGCT |
| vhOct4 | 134 | GTCCGAGGATCAACCC |
| vhSox2 | 124 | CCCGAAGTTTGCTGCG |
| vhKlf4 | 151 | GCTTGACGCAGTGTCT |
| vhc-Myc | 144 | CGCAGTAGAAATACGGCT |
