# Supplementary material for: Production of Embryonic and Fetal-Like Red Blood Cells from Human Induced Pluripotent Stem Cells
Source: PLoS One. 2011 Oct 13;6(10):e25761. doi: 10.1371/journal.pone.0025761 (PMC3192723; doi:10.1371/journal.pone.0025761)
Supplement: Table S5 — RT-PCR primers for endogenous genes. For each gene, top row is forward primer, bottom row is reverse primers. Sizes of the amplicons are in bp. (DOCX) [file pone.0025761.s011.docx]

**Table S5: RT-PCR primers for endogenous genes**

| **Gene** | **Amplicon** | **Seq.** |
| --- | --- | --- |
| beta-2-M | 124 | AGCAAGGACTGGTCTTT |
|  |  | CTCGATCCCACTTAACTATCT |
|  |  |  |
| GAPDH |  | CTATAAATTGAGCCCGCAG |
|  |  | AAATCCGTTGACTCCGA |
|  |  |  |
| hKlf4 | 125 | TGCATGCCAGAGGAGC |
|  |  | GCCTTGAGATGGGAACT |
|  |  |  |
| hc-Myc | 118 | GCGAACACACAACGTC |
|  |  | GATAACTACCTTGGGGGC |
|  |  |  |
| Nanog |  | GGCGGTAATGTTGGACATGAGCGAAT |
|  |  | CTGAAGCTTCCCGGGGGTACCGAAT |
|  |  |  |
| hNanog | 90 | TCCTTGCAAATGTCTTCTGCT |
|  |  | CAGGGCTGTCCTGAATAAGC |
|  |  |  |
| hOct4 | 250 | GACCCCTTCATTGACCTCAAC |
|  |  | CTTCTCCATGGTGGTGAAGA |
|  |  |  |
| hSox2 | 96 | ATGCACCGCTACGACGTGA |
|  |  | GCGAGTAGGACATGCTGTAGG |
|  |  |  |
| Pax6 | 112 | CCCCACATATGCAGACACAC |
|  |  | TCACTTCCGGGAACTTGAAC |
|  |  |  |
| Gata 6 | 213 | CCATGACTCCAACTTCCACC |
|  |  | ACGGAGGACGTGACTTCGGC |
|  |  |  |
| CDX-2 | 563 | GAACCTGTGCGAGTGGATGCG |
| CDX-2 |  | GGTCTATGGCTGTGGGTGGGAG |
|  |  |  |
| Brachyury | 208 | AGAGCCTGCAGTACCGAGTG |
| Brachyury |  | AGACACGTTCACCTTCAGCA |
